# Kelulut Honey as an Alternate Source of Carbo-Loading in Abdominal Surgery Involving the Digestive System: A Randomised Blinded Comparative Study

**DOI:** 10.21315/mjms-06-2024-419

**Published:** 2025-04-30

**Authors:** Karthik Krishnan, Siti Khatijah Abdul Razak, Mohd Zulkifli Mustafa, Najib Majdi Yaacob, Zalina Zahari, Mohd Shahrulsalam Mohd Shah, Andee Dzulkarnaen Zakaria

**Affiliations:** 1 Department of Surgery, School of Medical Sciences, Universiti Sains Malaysia, Health Campus, Kelantan, Malaysia; 2 Department of Neurosciences, School of Medical Sciences, Universiti Sains Malaysia, Health Campus, Kelantan, Malaysia; 3 Biostatistics and Research Methodology Unit, School of Medical Sciences, Universiti Sains Malaysia, Health Campus, Kelantan, Malaysia; 4 Faculty of Pharmacy, Universiti Sultan Zainal Abidin, Besut Campus, Besut, Terengganu, Malaysia

**Keywords:** Carbo-loading, kelulut, honey, insulin resistance, enhanced recovery after surgery, ERAS

## Abstract

**Background:**

Enhanced Recovery After Surgery (ERAS) is a multimodal perioperative care programme aimed at expediting recovery, reducing complications and minimising hospital stay by mitigating the metabolic stress response to surgery. One key shift in ERAS protocols is the replacement of traditional overnight fasting with carbohydrate-loading. Prolonged fasting increases insulin resistance, reduces cellular glucose uptake and glycogen formation, and may lead to hyperglycaemia. While maltodextrin is commonly used for carbohydrate-loading, it has a high glycaemic index and limited nutritional value. Kelulut Honey, a natural product rich in Trehalulose (a low-GI sugar), antioxidants, proteins, and vitamins may serve as a beneficial alternative.

**Methods:**

A randomised double-blind controlled trial involving 64 patients was conducted to evaluate the safety and efficacy of Kelulut Honey as a carbohydrate-loading agent in patients undergoing elective intra-abdominal surgery. Participants received either Kelulut Honey or commercial maltodextrin (Carborie^®^) according to ERAS protocol. Outcomes assessed included insulin resistance, residual gastric volume (RGV) and post-operative recovery parameters.

**Results:**

No significant difference was observed in blood glucose levels between the two groups. Average blood glucose at induction ranged from 5.3 to 5.6 mmol/L, with post-operative levels remaining below 10 mmol/L. Most participants passed flatus within 12 hours post-operatively, with over half ambulating and tolerating clear fluids within the same period. No significant differences were noted between groups in terms of complications, length of stay, pain control, or functional recovery. Intention-to-treat analysis confirmed comparable outcomes across all primary and secondary endpoints.

**Conclusion:**

Kelulut Honey is a safe and effective alternative to conventional carbohydrate drinks for pre-operative carbo-loading in abdominal surgery. It supports a modern shift away from the traditional surgical dogma of overnight fasting, with equivalent outcomes in glynaecmic control, RGV, and recovery compared to maltodextrin-based solutions.

## Introduction

Enhanced Recovery After Surgery (ERAS) is a well-established, evidence based multimodal, multidisciplinary approach to care for the surgical patient (1). The aim of the programme, as per its nomenclature, is to establish a set of protocols to conform to in order to facilitate recovery, attenuate the metabolic response from surgery, reduce complications and ultimately reduce the length of stay as well as establish an accelerated return to the physiological function of the patient (2, 3). Given that each year, approximately 234 million surgeries are performed throughout the world and over 90,000 surgeries were done by general surgery alone in Malaysia for the year 2011, it is imperative an evidence based system is utilised in order to accommodate this large number of patients in order to aid their recovery.

As ERAS involves a multimodal, multidisciplinary approach towards the surgical patient, there are many aspects and guidelines established for the implementation of ERAS. One particular modern care change is the conversion of the sacred surgical dogma of overnight fasting to carbo-loading, whereby the patient is subjected to the consumption of a carbohydrate-rich drink the evening prior to surgery and 2 hours prior to induction with anaesthesia. The doctrine of overnight fasting, or rather 6–8 hours of fasting, has long been the core principal for many anaesthesiologists and surgeons, given the rationale that this would reduce gastric acidity and volume, thereby reducing the risk of vomiting and gastric content aspiration (4). However, many guidelines have shown that there is no evidence that a shortened fast of 2–3 hours of oral fluids increases the risk of aspiration, regurgitation, or increased morbidity as compared with fasting overnight (5).

The challenge with fasting is that it increases insulin resistance. In line with acute-phase response, loss of lean body mass will attenuate the effects of prolonged fasting. So, in an insulin-resistant state, cell glucose uptake is reduced, and therefore, glycogen formation reduces, which means the liver and muscle glycogen storage are depleted. Subsequently, hyperglycaemia ensues due to the enhanced endogenous glucose production. Glucose control needs to be adequate to avoid the risk of surgical complications and mortality.

The benefit of carbo-loading is well documented and the evidence is overwhelming. Research shows that oral ingestion of 50 g of carbohydrates has been shown to release insulin, similar to that of a mixed meal. The current guideline advocates 100g of carbohydrate consumed the evening prior to surgery and another 50 g 2 hours before surgery. The first dose is consumed with 800 mL of water, and the subsequent dose with 400 mL.

Honey is nectar-based, carbohydrate-rich syrup produced by bees. Stingless Bee Honey, locally known as Kelulut Honey, is a natural product that contains sugar in the form of fructose and glucose. Successful domestication of *Haterotrigona itama* sp. since 2010 have promoted hygienic and GMP-HACCP certified honey production. This interest was further enhanced by the discovery of unique Trehalulose sugar that highlights the potential of the honey. The honey also has a proven role in facilitating wound healing and as an anti-inflammatory and anti-bacterial agent (6, 7, 8). The aim of this study is to evaluate the use of honey as an alternative to maltodextrin extract for carbo-loading in patients undergoing elective abdominal surgery involving the digestive system to achieve similar effects to conventional carbo-loading.

## Methods

### Research Design and Study Ethical Approval

The study was conducted at a single centre and was done in a randomised double-blinded control trial. It involved two groups: the Kelulut Honey (study arm) and the maltodextrin group (standard arm). All protocols are subjected to approval by the Human Ethical Committee at Universiti Sains Malaysia (approval number: USM/JEPeM/19110788), and written informed consent is acquired from the patients. The design of this trial aligns with recent multicenter studies evaluating carbohydrate-loading in major abdominal surgery (3).

### Study Population and Subject Criteria

Patients undergoing elective intra-abdominal surgery involving the digestive system, particularly the gastrointestinal and hepatobiliary system in the Department of Surgery, Hospital Universiti Sains Malaysia. Specifically, the organs involved include the oesophagus, stomach, and all parts of the colon, rectum, liver, gallbladder, and pancreas. Inclusion criteria are the adult (age ≥ 18) who undergoes **e**lective surgery with intra-abdominal involvement. Whereas, patients with known diabetic status, fasting glucose level > 7 mmol/L, ASA > 3, on steroid treatment, have a history of infection in the past 3 months, pre-operative unintentional weight loss > 10% of usual body weight within 6 months, emergency surgery, minor (age < 18) and known allergies to maltodextrin or honey are excluded from the study. Patients with change of mind and breach of protocols are withdrawn from the study.

### Operational Definition

#### Insulin Resistance

Post-operative impairment of response to insulin, leading to elevated glucose levels. Insulin resistance was reviewed and assessed six hours post-operative in view of the catabolic state response of the patient undergoing surgery.

#### Residual Gastric Volume

The amount of gastric volume aspirated from the patient via nasogastric aspiration after the induction of anaesthesia.

### Sample Size

Sample size were calculated for all analyses with type I error of 5%, type II error of 20% (80% power of study) and ratio of control to experimental group of 1. For comparison of blood glucose levels between patients receiving Kelulut Honey and Carborie pre-, intra, and post-operative period for 24 hours, the sample size was calculated using G*power software (test family: *t*-tests; statistical test: difference between two independent means). For comparison of residual gastric volume (RGV) between patients receiving Kelulut Honey and Carborie preoperatively immediately after induction of anaesthesia and prior to initiation of surgery, the sample size was calculated using n4Studies software (option: equivalence trial for continuous data). All calculations were done according to the previous methods (8). The largest sample size was taken as the final sample size, and after anticipating a 10% dropout, the corrected sample size was 32 patients per group. The total number of patients used in this study was, therefore, 64 patients.

### Randomisation and Masking

Random sequence generation of 64 random sequences was generated prior to patient recruitment in order to randomise participants into two equal-size groups: the Kelulut Honey group and the Carborie group. The sequence was generated by a statistician who is not involved in the process of recruitment, treatment and assessment of the patients. In order to avoid other researchers guessing the sequence, the random sequences were generated using permuted block randomisation with multiple random block size methods. The random sequence is then concealed using opaque sealed envelopes. Consecutive recruitment of participants and for each eligible and consented patient, a consecutive number from 1 to 64 was assigned. The main researchers then opened the envelope to reveal the patient’s group assignment. No matching of participants’ characteristics will be applied during the recruitment and randomisation process. Blinding: surgeons, anaesthetists, patients and outcome assessors were blinded to the treatment allocation. The blinding procedure is performed by preparing the beverage in an unlabelled amber-coloured bottle containing either Carborie or Kelulut Honey. The assessors are masked as they evaluate patients post-operatively during daily rounds without details regarding the liquid taken.

### Research Tool and Honey Preparation

This study used the data collection pro-forma form, a nasogastric tube for RGV determination and a Glucometer for glucose monitoring. Freshly prepared maltodextrin or Kelulut Honey mixed with water in 400 mL and 800 mL volumes in unlabelled ambercoloured bottles. For the purpose of this study, maltodextrin, known as Carborie^®^ from Valens^®,^ which is used in the standard arm, comes in powder form. For the initial dose on the evening prior to surgery, 100 g was used. The standard mixing instruction involves mixing 800 mL of water into the powder. This will provide 94.5 g of carbohydrate with 380 kcal. The next dose would be the morning dose 2 hours prior to anaesthesia, which will require mixing of 400 mL of water to 50VG of Carborie. This provides 47.3 g of carbohydrates and 190 kcals. For comparison, Carborie, an enteral carbohydrate polymer module used in the market for pre-operative carbohydrate-loading, has the following nutritional value, as shown in Table 1.

The assured quality Kelulut Honey was obtained from the Good Manufacturing Practice (GMP), Hazard Analysis Critical Control Point (HACCP) and ISO22000 certified local Kelulut Honey producer. The nutritional information of the Kelulut Honey sent for analyses at an accredited laboratory are as in Table 2.

For the honey group, 100 g of Kelulut Honey provides 69.7 g of carbohydrate. As such, a total of 135 g of honey provide 94.5 g of carbohydrate and 392 kcals for the initial dose in the evening prior to surgery. The subsequent dose in the morning, 2 hours prior to anaesthesia, will require 68 g of Kelulut Honey, which will provide 47.3 g of carbohydrates and 197 calories. The composition of honey was then freshly mixed with water to match 800 mL and 400 mL according as with the control arm.

### Study Protocols and Data Collection

For every patient enrolled in this study, the capillary blood glucose level is measured at the following intervals: hospital admission, upon arrival into the operating theatre, 1 hour after incision, at the end of surgery and later 24 hours post-operatively at regular intervals, at 6 am, 12 pm, 6 pm, 12 am. In the event the capillary blood glucose reading is 10.0 mmol/L or more that potentially hazardous, the insulin is administered according to the primary managing team decision. Patients in both control and study arms were given 800 mL of treatment solution at 8 pm the evening before surgery and 400 mL of the solution 2 hours before anaesthesia. Following induction and subsequent intubation, the anaesthetist will insert a nasogastric tube and measure the RGV via aspiration with a syringe. This value is recorded by both the anaesthetist and the surgeon later in the operative notes.

The patients will undergo surgery as per routine practice in Hospital Universiti Sains Malaysia. However, no corticosteroid administration can be administered. Post-operatively, only enteral feeding is allowed. Artificial nutrition, either enteral or parenteral, is not allowed. Enteral artificial nutrition is defined as feeding via feeding tubes such as Ryles tube or gastrostomy and parenteral artificial nutrition would be Total Parenteral Nutrition (TPN).

Antibiotic prescription, empirical or therapeutic, is left to the attending team to decide based on the standard of care. The duration of patient involvement in this study is until day four after surgery or on the day of discharge, whichever comes later. Should the patient return to the hospital after discharge but within 4 days from surgery, any complication that arose is included in the monitoring of this study.

Apart from RGV and insulin resistance, post-operative outcomes were also recorded. This included severity of complication based on the Dindo-Clavien classification system, post-operative unexpected antibiotic use, post-operative ICU admission, post-operative time for ambulation, post-operative time for flatus/ bowel function, length of hospital stay, and post-operative additional analgesia requirement (beyond initial operative plan). All data collection was recorded from the bed head ticket and pro-forma, which was then transferred to an electronic database.

### Statistical Analysis

After the patients have been randomised into their appropriate groups, an objective data analysis is conducted using the independent sample *t*-test. Secondary objective data assessment will be procured using the chi-squared test. Blood sugar level analysis between patients receiving Kelulut Honey and Carborie from induction to 24-h post-operatively was analysed using the Repeated Measure Analysis of Variance (RM ANOVA). The assumption of normally distributed residuals was assessed using visual inspection of Q-Q plots and the Shapiro-Wilk test for each time point. The assumption of sphericity, which requires equal variances of the differences between repeated measures, was tested using Mauchly’s test of sphericity. A significant result (*P* < 0.05) indicates that the sphericity assumption is violated. When Mauchly’s test indicated a violation of sphericity (*P* < 0.001), the Greenhouse-Geisser correction was applied to adjust the degrees of freedom for the F-statistics in the analysis of the time and time-treatment interaction effects. Partial eta squared (η^2^) was calculated to determine the effect sizes for the main effects (time and treatment) and the interaction effect (time × treatment). Partial eta squared values were interpreted as follows: small (0.01), medium (0.06), and large (0.14). The analysis was conducted using R software version 4.3.1 in the R Studio environment.

## Result

### Patient Enrolment and Surgery

**Figure 1:**
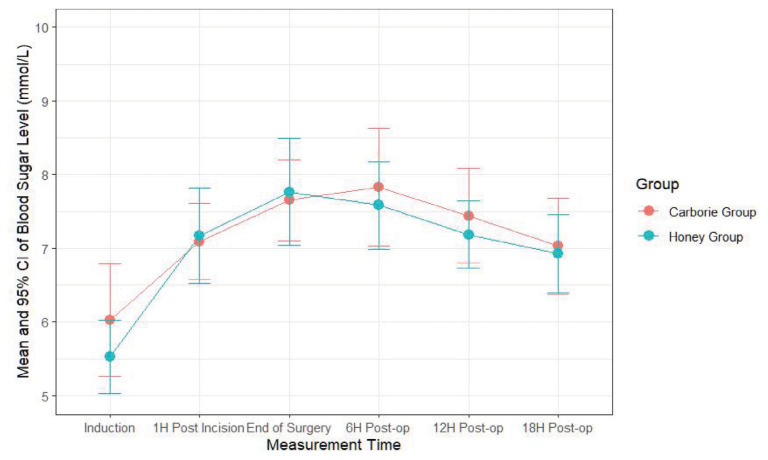
Profile plot for the blood sugar changes; the error bars represent the 95% confidence intervals of the means

A total of one hundred and twenty patients were assessed for eligibility. Seventy-two patients were found to fulfil the recruitment criteria, of which 64 patients consented to participate in this study and were randomised into either the Carborie group (A) or the Kelulut Honey group (B) (32 patients per group). One participant in the Carborie group was unable to complete the medication and therefore, was excluded from the study. The remaining 31 participants in the Carborie group and 32 participants in the Kelulut Honey group completed the oral intake of either Carborie or Honey and were included in the analysis (Figure 1). No adverse event was reported among all these study participants. The median operation duration was 107.5 (IQR = 65.5 to 148.2) minutes. There was no significant difference in the characteristics between both groups.

### Demographic Data

The characteristics of all the participants are summarised in Table 3. The mean age of study participants was 47.3 (SD = 16.6) years. The majority of the study participants are female (84.1%) with an average BMI of 27.2 (SD = 6.2) kg/m^2^. The average haematological and biochemical parameters were within the normal limit. More than half of the participants (54%) had at least one comorbidity. The majority of participants underwent surgical procedures involving the gallbladder (44%), followed by hernia (20.6%) and small bowel (14.3%).

### Insulin Resistance Analyses

RM ANOVA was analysed to compare the blood sugar levels between patients receiving Kelulut Honey and Carborie from induction to 24-h post-operatively. RM ANOVA analysis for the blood sugar levels indicates a significant time effect with no significant treatment- and time-treatment interaction effect (Table 4, Figure 1). The assumptions for repeated measures ANOVA were evaluated. Normality of the residuals which was assessed through visual inspection of residual histograms and Q-Q plots showed no significant deviations. Maunchly’s test of sphericity for the time × treatment interaction effect was significant (*P* < 0.001), indicating a violation in the sphericity assumption, and therefore, the Greenhouse-Geisser correction was applied to adjust the degrees of freedom.

The partial eta^2^ values were reported to assess the effect size of the RM ANOVA. It was utilised as a measure of the proportion of variance in the dependent variable that is explained by the independent variable. For time effect, the partial eta^2^ was 0.121, which indicates a medium effect size. This suggests that time accounted for a moderate proportion of the variance in blood glucose levels over the post-operative period. The relatively small partial eta^2^ for treatment and time-treatment interaction effects (0.002 and 0.004, respectively) suggests that these factors had minimal impact on the overall blood glucose levels when compared to the time effect. These results imply that while time significantly influences blood glucose levels, the type of treatment (Kelulut Honey vs Carborie) and the interaction between time and treatment have a negligible effect.

Within the group, changes of blood sugar levels (time effect) indicate a significant increment from induction to an hour post-incision, end of surgery, six hours post-op, and 12 hours post-op in the Carborie group. Significant increments in blood sugar from induction to an hour post-incision, end of surgery, six hours post-op, 12 hours post-op, and 18 hours post-op were observed in the Kelulut Honey group. There were no significant changes in the other time comparisons within both groups (Table 5).

The blood glucose levels observed in this study align with findings from nondiabetic patients undergoing major surgery under general anaesthesia, where glucose levels remained within a controlled range (5).

### RGV Analyses

The RGV comparison of the residual gastric volume between patients receiving Kelulut Honey and Carborie was done preoperatively immediately after induction of anaesthesia and prior to initiation of surgery. Measurement of RGV was made in all 63 participants. There was no significant difference in the RGV between both groups.

### Post-operative Outcomes

The post-operative outcomes, including complication, length of stay, pain control, and return to function between patients receiving Kelulut Honey and Carborie were compared. Complications, length of stay, pain control and return to function were evaluated post-operatively in all the study participants. The median length of stay was 4.0 (IQR = 3.0 to 5.0) days. Six (9.7%) require an increase in analgesia. The majority of the participants could pass flatus either < 6 h or between 6–12 h post-operatively. More than half of the participants were able to ambulate within < 12 h of surgery, and more than half were started on clear fluid < 6 h post-operatively. Comparison between groups indicates no significant difference in terms of complication, length of stay, pain control, and return to function. The findings are summarised in Table 7.[Table t6-06mjms3202_oa]

### Intention-to-Treat Analysis

**Figure 3:**
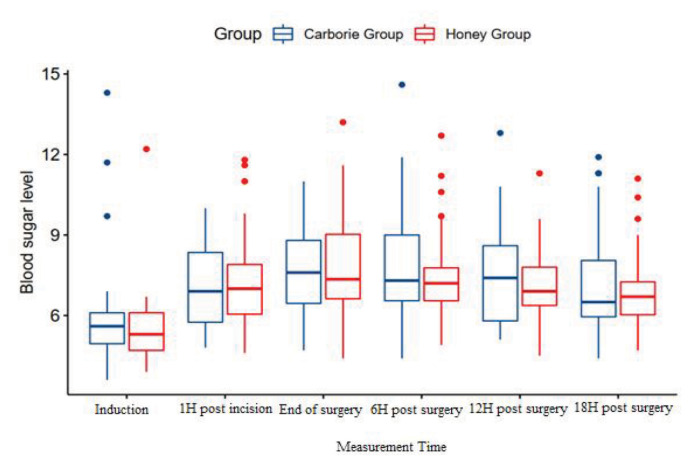
Comparison of blood glucose levels based on the intention-to-treat analysis

Intention-to-treat analysis was done according to blood glucose levels. Comparisons were made between patients receiving Kelulut Honey and Carborie in pre-, intra, and post-operative periods in 24 hours. Multiple imputations were performed to enable an intention-to-treat analysis. This analysis revealed no significant difference in the blood glucose levels of both groups throughout the measurement time (Table 7, Figure 3).

## Discussion

Maltodextrin is a polysaccharide sugar made from starch and commonly used to improve the texture of processed food and drinks. It is known to possess a high glycemic index with fewer beneficial nutrients. Kelulut Honey is a natural substance with known health benefits. It contains antioxidants, vitamins, minerals, fibre, phytochemicals, and protein (9). Availability of new ecosystem in Kelulut Honey production in Malaysia, including MyGAPam (Malaysian Good Agriculture Practice Apis/Meliponine), Malaysian Standard for Kelulut Honey (MS2673) as well as GMP, HACCP and ISO22000 certified production plant offer a new standard of honey. A newly recognised unique Trehalulose sugar that is abundance in Kelulut Honey (6) makes it possible for standardisation of the honey.

The study shows that there is no significant difference in the outcome of the effect of Kelulut Honey on insulin resistance and RGV when used as carbo-loading preoperatively when compared with Carborie. The gastric emptying of honey is similar to the findings from the previously referred study by Sudiyatmo et al. and Yagci (10, 11) and demonstrates rapid absorption and metabolic benefits (12). This shows that the Kelulut Honey is safe to be used for pre-operative carbo-loading at a volume of 800 mL the evening before surgery and 400 mL two hours prior to anaesthesia induction. As for the mean of gastric content among patients who underwent abdominal surgery, 18.46 mL with a standard deviation of 16.38 mL was reported.

As previously referenced, a favourable effect of pre-operative carbohydrate-loading on glucose metabolism and insulin resistance is well documented (12, 13), and given that there is no statistically significant difference between the two arms, it is deduced that a similar benefit can be acquired with carbo-loading utilising Kelulut Honey.

From the glucose monitoring analysis, at the point of induction, the blood glucose levels average around the normal glucose level. As postulated, there is a spike in blood glucose level an hour after incision and the elevation averages around 7 mmol/L for both standard and study arms, with the peak being around 9 mmol/L, which can be attributed to the metabolic response from surgery and the blood glucose control may indicate the avoidance of insulin resistance from carbo-loading (14). When the RM ANOVA analysis was performed for the blood sugar levels over time, it was noted there was a significant increment from induction to an hour post-incision, end of surgery, six hours post-op and 12 hours post-op in both Carborie and Honey groups. However, there was no significant difference between the time comparisons within both groups, as pictured by the graph, indicating the mean of 95% confidence interval for blood sugar over time in both groups. This implies that the effect of glucose control is similar in both groups, thereby having a similar effect on insulin resistance. A previous study reported that the standard deviation of blood glucose level among fasting patients who underwent abdominal surgery was 1.38 mmol/L (11).

The secondary outcome was intended to compare post-operative outcomes (feeding, length of stay, pain control and return to function, severity of complications) for patients receiving Carborie and Kelulut Honey also shows comparable outcomes between the two groups. More than half of the patients were able to ambulate within 12 hours from surgery, and more than half were started on clear fluids less than 6 hours post-operatively. The majority of participants could pass flatus within 12 hours post-operatively. The average length of stay between both groups was about 4 days. Comparison between both groups indicates no significant difference in terms of post-operative outcome analysed.

Data analysis shows that the primary and secondary objectives and outcomes of both Kelulut Honey and Carborie are not significantly different. Kelulut Honey is safe to be used as carbo-loading for patients undergoing elective abdominal surgery involving the digestive system and the outcomes are favourable and similar to Carborie.

Nevertheless, this study is confined to only a single centre, and the variety of patients involved is limited by the local population and the local expertise of the treating physician. Given the location of Kubang Kerian, Kelantan, the local population is largely made up of the Malay populace, and the composition of patients from different races is minimal.

Furthermore, this study only involved patients undergoing elective abdominal surgery involving the digestive tract, and even then, there was high heterogeneity in the types of surgery that were performed, as there were many types of surgery involving different target organs within the abdomen.

Another limitation was that only a specific batch of Kelulut Honey produced from a single farm was used for this purpose. While using the same batch of honey allowed standardisation, it is not possible to predict the outcome from Kelulut Honey produced from a similar species of bee from different geographical areas. Likewise, other types of honey from different species of bees, such as typical honeybees, were not explored in this analysis either.

## Conclusion

Kelulut Honey is safe to use as an alternative to conventional carbo-loading in patients undergoing abdominal surgery involving the digestive system, and its efficacy is shown to be similar to the commercially available product, Carborie. Analysis pertaining to RGV, insulin resistance, length of hospital stay, complications, return to function and pain control between patients receiving either Carborie or Honey are similar. Given that carbo-loading with a carbohydrate drink prior to surgery at fixed intervals is now propagated by ERAS, the data analysis concludes that a similar outcome can be expected from using Kelulut Honey.

## Figures and Tables

**Figure 2:**
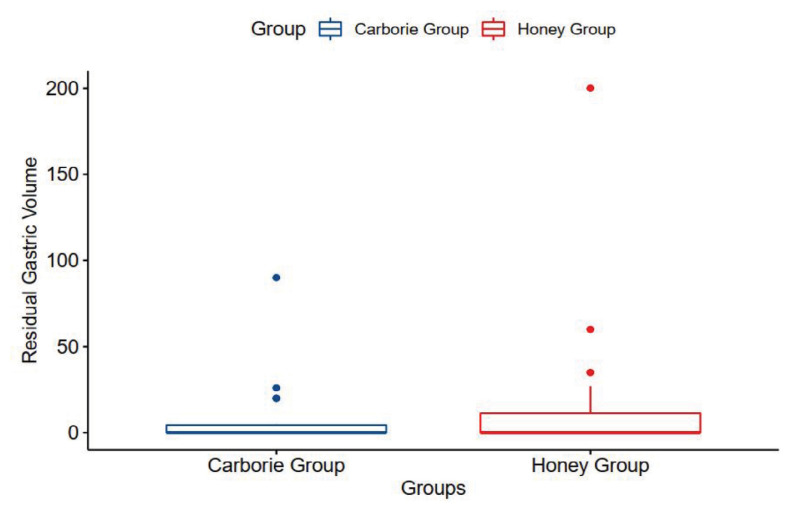
Box plot showing the comparison of the residual gastric volume between groups (graph should indicate RGV volume in mL)

**Table 1:** Nutritional value for Carborie

Nutritional information	Evening dose (800 mL)	Morning dose (400 mL)
Energy	380 kcal	190 kcal
Protein	0	0
Carbohydrate	94.5 g	47.3 g
Fat	0 g	0 g
Calcium	25 mg	0.5 mg
Sodium	70 mg	1.4 mg
Iron	0 mg	0 mg
Potassium	1 mg	0.5 mg
Chloride	150 mg	75 mg
Phosphorus	8 mg	4 mg
Osmolality	133 mOsm/kg	133 mOsm/kg

**Table 2:** Nutritional information of the Kelulut Honey

Parameter	Analysis results	Unit
Energy from fat	10.0	kcal/100 g
Fructose	22.5	g/100 g
Glucose	23.6	g/100 g
Hydroxylmethylfurfural (HMF)	2.9g	mg/kg
Sucrose	ND (< 0.5)	g/100 g
Trehalulose	53.9	g/100 g
Calcium	9.0	mg/100 g
Carbohydrate	69.7	g/100 g
Crude protein	0.5	g/100 g
Dietary fibres	ND (< 0.5)	g/100 g
Energy	291	kcal/100 g
Fat	1.1	g/100 g
Iron	ND (< 0.5)	mg/kg
Lead	ND (< 0.5)	mg/kg
Sodium	8.9	mg/100 g
Zinc	ND (< 0.5)	mg/kg
pH	3	-
Moisture content	27	%

Note: ND = not detected

**Table 3:** Characteristics of the overall study participants and comparison according to group

Variables	*N*	Overall, *N* = 63	Carborie group (*n* = 31)	Honey group (*n* = 32)	*P*-value*
Age (year)	63	47.3 (16.6)	46.6 (16.1)	47.9 (17.3)	0.760

Gender	63				0.509
Female		53 (84.1%)	25 (80.6%)	28 (87.5%)	
Male		10 (15.9%)	6 (19.4%)	4 (12.5%)	

BMI (kg/m^2^)	63	27.2 (6.2)	27.6 (6.4)	26.7 (6.0)	0.566

Hb (g/dL)	63	13.2 (1.6)	13.3 (1.8)	13.1 (1.5)	0.602

WBC	63	8.1 (2.2)	8.5 (2.4)	7.8 (1.9)	0.222

Platelet	63	291.9 (87.7)	308.8 (85.9)	275.5 (87.7)	0.134

Urea (mmol/L)	63	4.3 (1.3)	4.4 (1.3)	4.1 (1.4)	0.363

Creatinine (umol/L)	63	74.9 (18.8)	79.3 (16.3)	70.6 (20.3)	0.067

Na (mmol/L)	63	136.8 (2.3)	136.7 (2.0)	136.9 (2.6)	0.695

K (mmol/L)	63	4.1 (0.5)	4.1 (0.4)	4.1 (0.6)	0.865

Albumin (g/L)	61	42.8 (4.6)	43.4 (4.3)	42.2 (4.9)	0.300

TP (g/L)	61	72.4 (7.5)	72.3 (7.5)	72.4 (7.6)	0.978

TB (umol/L)	61	10.0 (7.0, 15.0)	10.0 (7.0, 12.0)	10.0 (7.0, 15.0)	0.879

Comorbids	63	34 (54.0%)	18 (58.1%)	16 (50.0%)	0.521

# Comorbid	63				0.818
0		29 (46.0%)	13 (41.9%)	16 (50.0%)	
1		21 (33.3%)	12 (38.7%)	9 (28.1%)	
2		10 (15.9%)	5 (16.1%)	5 (15.6%)	
3		3 (4.8%)	1 (3.2%)	2 (6.2%)	

HPT	63	13 (20.6%)	5 (16.1%)	8 (25.0%)	0.384

Diabetes	63	0 (0.0%)	0 (0.0%)	0 (0.0%)	> 0.95

IHD	63	4 (6.3%)	1 (3.2%)	3 (9.4%)	0.613

Malignancy	63	10 (15.9%)	6 (19.4%)	4 (12.5%)	0.509

ASA	63				0.609
1		25 (39.7%)	11 (35.5%)	14 (43.8%)	
2		37 (58.7%)	19 (61.3%)	18 (56.2%)	
3		1 (1.6%)	1 (3.2%)	0 (0.0%)	

Target_organ	63				0.841
Liver		4 (6.3%)	2 (6.5%)	2 (6.2%)	
Gallbladder		28 (44.4%)	12 (38.7%)	16 (50.0%)	
Stomach		2 (3.2%)	2 (6.5%)	0 (0.0%)	
Small bowel		9 (14.3%)	5 (16.1%)	4 (12.5%)	
Colon		6 (9.5%)	3 (9.7%)	3 (9.4%)	
Rectum		1 (1.6%)	1 (3.2%)	0 (0.0%)	
Hernia		13 (20.6%)	6 (19.4%)	7 (21.9%)	

Surgeon	63				> 0.95
Registrar		5 (7.9%)	2 (6.5%)	3 (9.4%)	
Surgeon		37 (58.7%)	18 (58.1%)	19 (59.4%)	
Consultant		21 (33.3%)	11 (35.5%)	10 (31.2%)	

Op duration (min)	62	107.5 (65.5, 148.2)	86.0 (65.5, 131.2)	120.0 (76.2, 162.5)	0.352

Notes: Median (IQR) are presented for TB and Op duration; all other numerical variables are presented as Mean (SD); all categorical variables are presented as *n* (%);

* *P*-value for two-sample *t*-test, Fisher’s exact test, Mann-Whitney test, and Pearson’s chi-squared test;

Hb = Haemoglobin; WBC = White Blood Cell count; Na = Sodium; K = Potassium; TP = Total Protein; TB = Total Bilirubin; HPT = Hypertension; IHD = Ischaemic Heart Disease; ASA = American Society of Anesthesiologists physical status classification

**Table 4:** Statistical values of the test effects (time, treatment, and time-treatment interaction effect) for the blood sugar levels

Test effects	F-statistics (df)	*P*-value	Partial eta^2^
Time	15.95 (5, 305)	< 0.001	0.121
Treatment	0.25 (1, 61)	0.623	0.002
Time*Treatment	0.41 (3.28, 200.18)*	0.764	0.004

Note:

* Assumption of compound symmetry is violated (Mauchly’s test of sphericity *P*-value < 0.001)

**Table 5:** Changes of blood sugar levels within each group based on time (time effect)

Comparison	Carborie group		Kelulut Honey group	
	MD (95% CI)	*P*-value	MD (95% CI)	*P*-value
Induction – 1 h post-incision	−1.07 (−1.74, −0.4)	0.042	−1.63 (−2.21, −1.06)	< 0.001
Induction – End of surgery	−1.62 (−2.36, −0.88)	0.002	−2.23 (−2.87, −1.59)	< 0.001
Induction – 6 h post-op	−1.80 (−2.79, −0.81)	0.012	−2.05 (−2.65, −1.45)	< 0.001
Induction – 12 h post-op	−1.41 (−2.25, −0.57)	0.027	−1.65 (−2.27, −1.03)	< 0.001
Induction – 18 h post-op	−1.00 (−1.83, −0.18)	0.284	−1.39 (−2.05, −0.74)	0.002
1 h post-incision – End of surgery	−0.55 (−0.93, 0.18)	0.810	−0.60 (−0.97, −0.23)	0.039
1 h post-incision – 6 h post-op	−0.73 (−1.58, 0.12)	> 0.95	−0.42 (−1.29, 0.46)	> 0.95
1 h post-incision – 12 h post-op	−0.35 (−1.08, 0.39)	> 0.95	−0.02 (−0.78, 0.75)	> 0.95
1 h post-incision – 18 h post-op	0.06 (−0.62, 0.75)	> 0.95	0.24 (−0.57, 1.06)	> 0.95
End of surgery – 6 h post-op	−0.18 (−1.02, 0.67)	> 0.95	0.18 (−0.77, 1.13)	> 0.95
End of surgery −126 h post-op	0.21 (−0.59, 1.01)	> 0.95	0.58 (−0.23, 1.39)	> 0.95
End of surgery – 18 h post-op	0.62 (−0.11, 1.35)	> 0.95	0.84 (0.04, 1.63)	0.590
6 h post-op – 12 h post-op	0.39 (−0.23, 1.01)	> 0.95	0.40 (−0.11, 0.91)	> 0.95
6 h post-op – 18 h post-op	0.80 (0.02, 1.57)	0.676	0.66 (−0.12, 1.43)	> 0.95
12 h post-op – 18 h post-op	0.41 (−0.19, 1.01)	> 0.95	0.26 (−0.3, 0.81)	> 0.95

Notes: MD=mean difference; CI=confidence intervals; Test of within subject effect for the Carborie group: F(3.42, 102.46) = 6.09, *P* < 0.001; Test of within subject effect for the Kelulut Honey group: F(2.74, 84.92) = 10.53, *P* < 0.001

**Table 6 t6-06mjms3202_oa:** Comparison of RGV between groups.

Variable	*N*	Overall, *N* = 63	A, *n* = 31	B, *n* = 32	*P*-value*
Residual gastric volume	63	0.0 (0.0, 9.5)	0.0 (0.0, 4.5)	0.0 (0.0, 11.2)	0.152

Notes: All measurements are presented as median (IQR);

* Mann-Whitney test

**Table 7:** Complication, length of stay, pain control, and return to function

Variable	*N*	Overall, *N* = 63	A, *N* = 31	B, *N* = 32	*P*-value*
Complications	63	4 (6.3%)	2 (6.5%)	2 (6.2%)	>0.95

Length of stay (days)	63	4.0 (3.0, 5.0)	4.0 (3.0, 5.5)	4.0 (3.0, 5.0)	0.604

Analgesia increase	63	6 (9.5%)	2 (6.5%)	4 (12.5%)	0.672

Flatus (hour)	63				0.734
< 6 h after surgery		20 (31.7%)	8 (25.8%)	12 (37.5%)	
> 6 h after surgery		20 (31.7%)	11 (35.5%)	9 (28.1%)	
> 12 h after surgery		13 (20.6%)	6 (19.4%)	7 (21.9%)	
> 24 h after surgery		10 (15.9%)	6 (19.4%)	4 (12.5%)	

Ambulation (hour)	63				0.282
< 6 h after surgery		11 (17.5%)	4 (12.9%)	7 (21.9%)	
> 6 h after surgery		23 (36.5%)	15 (48.4%)	8 (25.0%)	
> 12 h after surgery		15 (23.8%)	6 (19.4%)	9 (28.1%)	
> 24 h after surgery		14 (22.2%)	6 (19.4%)	8 (25.0%)	

Clear fluid feeding (hour)	63				0.414
< 6 h after surgery		37 (58.7%)	15 (48.4%)	22 (68.8%)	
> 6 h after surgery		16 (25.4%)	10 (32.3%)	6 (18.8%)	
> 12 h after surgery		4 (6.3%)	2 (6.5%)	2 (6.2%)	
> 24 h after surgery		6 (9.5%)	4 (12.9%)	2 (6.2%)	

Nourishing fluid feeding (hour)	63				0.421
< 6 h after surgery		20 (31.7%)	7 (22.6%)	13 (40.6%)	
> 6 h after surgery		20 (31.7%)	10 (32.3%)	10 (31.2%)	
> 12 h after surgery		14 (22.2%)	8 (25.8%)	6 (18.8%)	
> 24 h after surgery		9 (14.3%)	6 (19.4%)	3 (9.4%)	

Normal diet (hour)	63				0.253
< 6 h after surgery		13 (20.6%)	4 (12.9%)	9 (28.1%)	
> 6 h after surgery		14 (22.2%)	9 (29.0%)	5 (15.6%)	
> 12 h after surgery		21 (33.3%)	9 (29.0%)	12 (37.5%)	
> 24 h after surgery		15 (23.8%)	9 (29.0%)	6 (18.8%)	

Notes: All variables are presented as either *n* (%) or Median (IQR);

* *P*-value for Fisher’s exact test, Mann-Whitney test and Pearson’s chi-squared test
